# Global gene regulation during activation of immunoglobulin class switching in human B cells

**DOI:** 10.1038/srep37988

**Published:** 2016-11-29

**Authors:** Youming Zhang, David J. Fear, Saffron A. G. Willis-Owen, William O. Cookson, Miriam F. Moffatt

**Affiliations:** 1 Molecular Genomics and Genetics Group, National Heart and Lung Institute, Imperial College, London SW3 6LY, UK; 2 Medical Research Council and Asthma UK Centre in Allergic Mechanisms of Asthma, King’s College London, London, United Kingdom

## Abstract

Immunoglobulin class switch recombination (CSR) to IgE is a tightly regulated process central to atopic disease. To profile the B-cell transcriptional responses underlying the activation of the germinal centre activities leading to the generation of IgE, naïve human B-cells were stimulated with IL-4 and anti-CD40. Gene expression and alternative splicing were profiled over 12 days using the Affymetrix Human Exon 1.0 ST Array. A total of 1,399 genes, forming 13 temporal profiles were differentially expressed. *CCL22* and *CCL17* were dramatically induced but followed a temporal trajectory distinct from classical mediators of isotype switching. *AICDA*, *NFIL3*, *IRF4*, *XBP1* and *BATF3* shared a profile with several genes involved in innate immunity, but with no recognised role in CSR. A transcription factor *BHLHE40* was identified at the core of this profile. B-cell activation was also accompanied by variation in exon retention affecting >200 genes including *CCL17*. The data indicate a circadian component and central roles for the Th2 chemokines CCL22 and CCL17 in the activation of CSR.

IgE levels are under tight regulatory control, with IgE possessing the shortest half-life and being the least abundantly expressed of all immunoglobulin isotypes^1^. IgE underlies type I hypersensitivity and raised IgE levels are seen in increasingly prevalent atopic diseases including asthma, atopic dermatitis and allergic rhinitis. Antibody isotype is determined by the constant region genes of the heavy Ig chain locus (C_H_), which confer distinct effector properties to the immunoglobulin such as Fc receptor specificity, complement activation, stability, ability to transcytose and thereby also tissue distribution. IgE is produced when B cells are stimulated to undergo Immunoglobulin class switch recombination (CSR) in response to antigen stimulation and co-stimulatory signals, replacing the constant region genes at the heavy chain locus (initially Cμ and Cδ) with those encoding IgE (Cε). For a B cell to successfully undergo CSR, a number of cellular processes including, proliferation, control of apoptosis, DNA recombination and cell differentiation must be coordinated. These processes usually occur in the germinal centres of secondary lymphoid organs or local tissues following antigen encounter in the presence of T cell help. While the core mechanisms of the germinal centre activities leading to IgE are well established, the fine-scale transcriptional machinery underlying the production and dynamic regulation of IgE remains relatively unknown^2^ despite its clinical relevance.

In this study, we have simultaneously monitored the changes in gene expression both in terms of coarse-scale gene abundance and fine-scale exon retention that accompany the activation and progression of class switching in human B cells. Naïve B cells were stimulated *in vitro* with IL-4 and anti-CD40 signals that mimic the T cell help received in the germinal centre, inducing a strong activation of NF-kB leading to a proliferative burst and CSR to IgE and IgG^3^. This co-stimulation signal was therefore applied here to provide a controlled window on the molecular pathways that regulate these germinal centre processes underlying IgE production in human B cells^4^.

## Results

### Temporal variation in and patterns of global gene expression during activation of CSR

At a 5% false discovery rate (FDR) a total 1,399 genes (transcript clusters, TCs) attained significance for differential expression in one or more of the assayed time windows (Figure S1). Consistent with known biology^5^,^6^ these differentially expressed genes were most significantly enriched for biological process terms relating to *cell cycle* (GO:0007049, *P*-value 1.50E-27, Fold Enrichment 2.26) and localisation within the *chromosome* cellular component (GO:0005694, *P*-value 5.51E-20, fold enrichment 2.46). The most enriched molecular function was *ATP binding* (GO:0005524, *P*-value 1.54E-09, fold enrichment 1.55).

**Figure 1:**
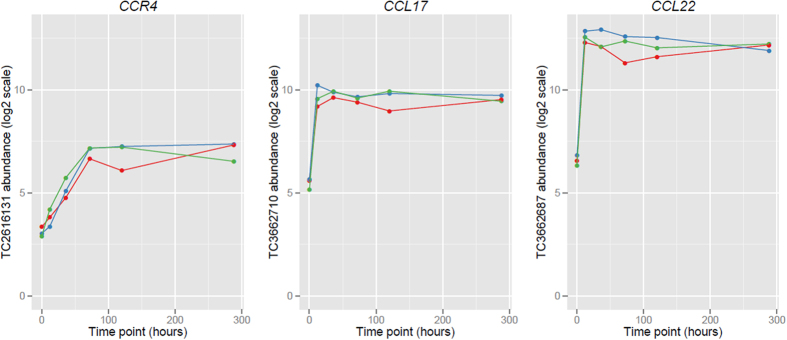
Expression of *CCR4* and its ligands *CCL22*, and *CCL17* during the activation of CSR. Abundance is displayed on a log2 scale. Results of triplicates are shown. Time point is in hours. Abbreviations: Transcript Cluster (TC).

The most significant differential expression, with an adjusted *P*-value of 4.39E-07, was attained at TC 3662687, the potent Th2 and regulatory T-cell attracting chemokine gene, *CCL22*. CCL22 and CCL17 are both ligands for the chemokine receptor CCR4^7^ and all three of these targets fall within the top twenty genes differentially expressed during the activation of immunoglobulin class switching in human activated B cells (Table 1, Fig. 1). *CCL22* and *CCL17* were potently induced during the early phase of activation, with a substantial 63.95 fold increase for *CCL22* during the first 12 hours, and 18.33 fold increase for *CCL17* during the same period. Both *CCL22* and *CCL17* are NF-kappa B (NF-kB) target genes, highlighting a central role for the NF-kB pathway in the activation of CSR. The top differentially expressed genes also contained another NF-kB target gene the TNF receptor associated factor (*TRAF1*)^8^, as well as the cytokine receptor *IL17RB* which mediates the activation of NF-kB.

Other less well characterised differentially expressed genes include the non-enzymatic replication factor *MCM10* (TC 3235789), kinesin family member 14 (*KIF14*), part of a proliferation signature seen during early B-cell development^9^ (TC 2450345), and a protein regulator of cytokinesis (*PRC1*) (TC 3639031, Table 1). PRC1 is a known associative protein of KIF14^10^, acting together to promote cell division, and all three of these genes have reported mutations and/or clinically significant variation in abundance in various cancers^11^,^12^. These cytokinesis and proliferation related transcriptional changes may have implications for the efficiency of class switching, as well as the regulation of isotype class^13^,^14^.

**Figure 2:**
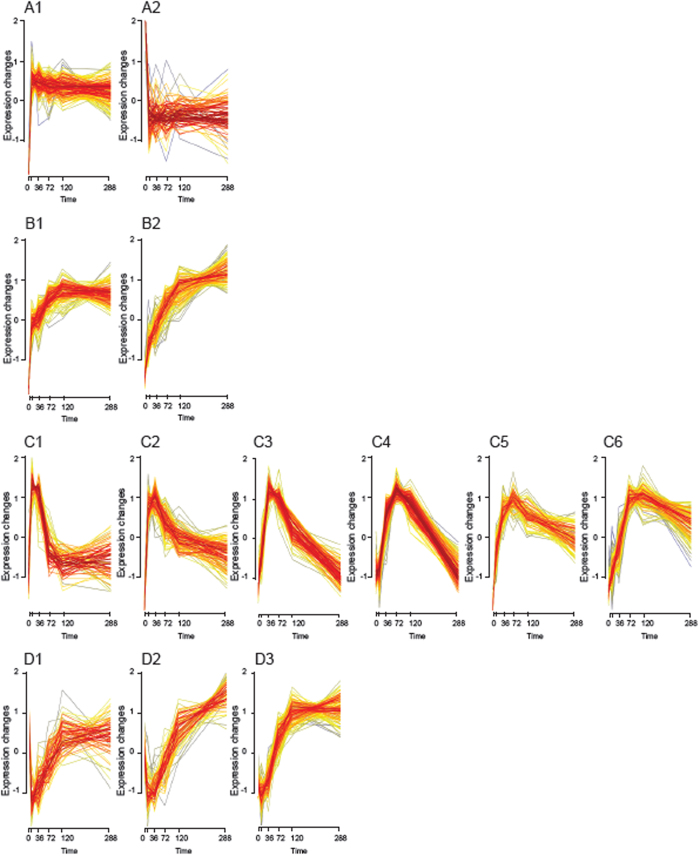
Temporal expression patterns exposed by soft clustering of differentially expressed genes. Soft clusters of genes differentially expressed during activation of CSR. Genes are colour-coded according to their cluster membership values. Genes exhibiting the highest membership values are shown in red. Pattern class (**A**) quasi-on/off; Pattern class (**B**) graduated induction; Pattern class (**C**) Transient induction; Pattern class (**D**) Transient down regulation.

Next the complexity of variation in transcription amongst the 1,399 genes differentially expressed during the activation of CSR was reduced to 13 central temporal transcription profiles using fuzzy c-means clustering (Fig. 2 and S2, Table 2). This unsupervised classification technique clusters genes that exhibit temporal coordination and reveals cluster cores; tightly co-expressed genes potentially under shared regulatory control. Genes possessing highly correlated expression profiles are more likely to be bound by a common transcription factors^15^. As such, clusters were assessed for an over-representation of individual Transcription Factor Binding Sites (TFBS) amongst members, demonstrating significant enrichment in 3 clusters (Table 2). Clusters could also be at least partially differentiated by biological process through patterns of enrichment for gene ontology terms (Table 2). Inspection of the identified clusters revealed on/off phenomena (Clusters A1 and A2), as well as groups of genes showing graduated sustained induction (Clusters B1 and B2), transient induction (Clusters C1–C6) and transient down-regulation (Clusters D1–D3).

**Figure 3:**
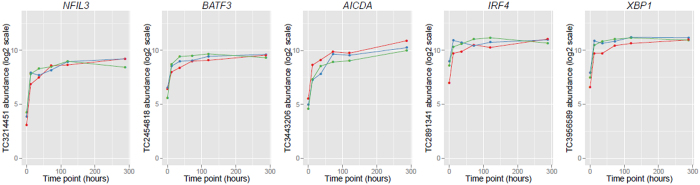
Expression of five key CSR genes over the 288 hour time course following IL-4 and anti-CD40 stimulation. Abundance is displayed on a log2 scale. Results of triplicates are shown. Time point is in hours. Abbreviations: Transcript Cluster (TC).

**Figure 4:**
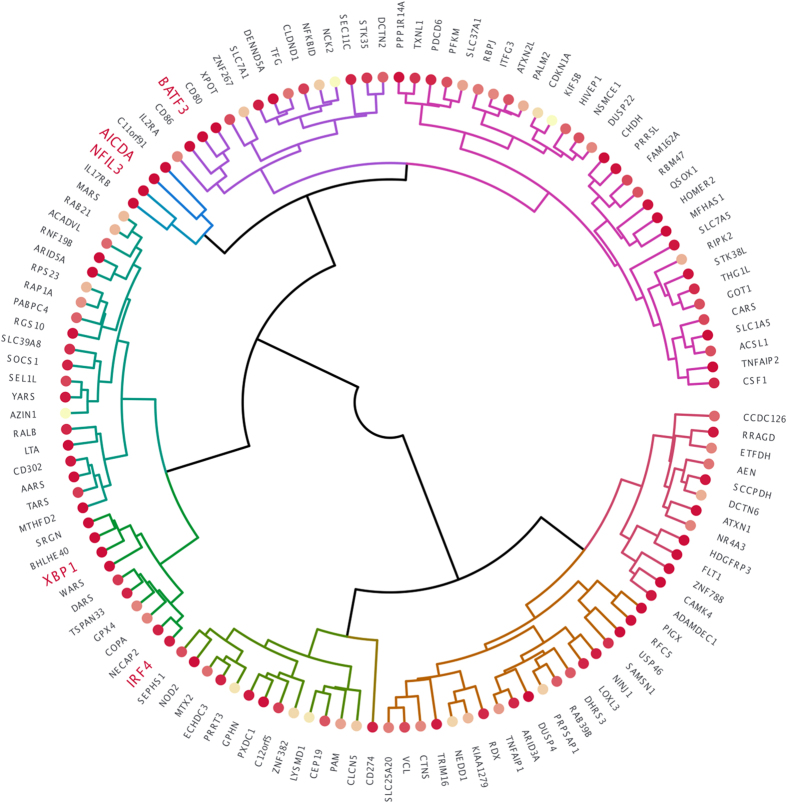
Fine-scale substructure within temporal transcription cluster B1. Figure 4 is a circulized dendrogram of temporal transcription cluster B1. Branches are coloured according to their groupings using a cut height of 4. Colour density at the branch terminal nodes reflects the significance of differential expression, with the smallest *P*-values yielding the darkest nodes. For ease of interpretation only the first Affymetrix gene annotation is shown. Five genes with well-established roles in class switching and germinal centre cell function are highlighted in red text.

Five genes with well-established roles in class switching and germinal centre cell function were found to cluster together in a single temporal profile representing genes that are rapidly activated during the stimulation time course, cluster B1, containing 126 members (Fig. 3). Cluster B1 contained the lymphocyte specific transcription factor interferon regulatory factor 4 (*IRF4*)^16^, the DNA deaminase *AICDA* encoding AID^17^, the transcriptional activator and regulator of the unfolded protein response *XBP1*, the CSR-related transcription factor *BATF3*^18^,^19^, and the B-cell intrinsic transcriptional regulator (*NFIL3*) that was recently identified as a key regulator of IgE class switching in mice^20^. All five genes were highly differentially expressed during the activation time course (Fig. 4; *IRF4*, TC 2891341, F = 10.68, *P* = 3.37E10-03; *AICDA*, TC 3443206, F = 40.21, *P* = 9.40E10-06; *XBP1*, TC 3956589, F = 29.25, *P* = 3.22E10-05; *BATF3*, TC 2454818, F = 33.99, P = 1.84E-05; *NFIL3*, transcript cluster 3214451, F = 65.55, *P* = 2.08E-06).

Fine-scale structure within cluster B1 was revealed by complete linkage hierarchical clustering (Fig. 4), indicating that *AICDA’s* closest neighbours are *C11orf91* (TC 3368707); *NFIL3* (TC 3214451) a key regulator of IgE production, airway hyper-responsiveness and effector T-cell responses^21^,^22^; and *IL17RB* (TC 2624565), a central component of NF-kB activation.

Other members of temporal cluster B1 included several genes previously implicated in innate immune function through allelic association. These include Lymphotoxin alpha, *LTA* (TC 2902407), variants of which have been associated with asthma^23^; the innate immune receptor *NOD2* (TC 3660175) which has been associated with several atopy related traits^24^,^25^ and the negative regulator of lipopolysaccharide (LPS) signalling *SOCS1* (TC 3680213), variants of which have been associated with total serum IgE^26^. The impact these allelic variants have on CSR efficiency, B cell differentiation and IgE production may be an area worthy of future exploration.

Cluster B1 showed a rapid and strong induction at 12 hours with continued increments in abundance up to 120 hours after which it plateaued. The cluster was significantly enriched for motifs binding the transcription factors *RSRFC4 (P*-value 9.32E-03) and *STAT (P*-value 1.71E-02). The gene statistically most central to, or representative of cluster B1 was the transcription factor *BHLHE40. BHLHE40* is a primary target of the vitamin D receptor^27^, regulated by environmental signals and conferring downstream effects on the cell cycle, cellular differentiation and the mammalian molecular clock^28^. Vitamin D deficiency is a growing area of interest in the development and modulation of asthma and allergies^29^. Given the presence of several known regulators of B-cell germinal centre function in cluster B1, cluster membership and within-cluster proximity may provide a useful metric for prioritising differentially expressed genes for further analysis.

Similar to cluster B1, cluster A1 (153 members) demonstrated a potent early induction at 12 hours but rapidly stabilised after this time point, resembling an ‘on-switch’ phenomenon. Central to this cluster, and exhibiting the highest membership values, were *CCL22* and *CCL17* (0.97 and 0.96 respectively); two of the top three genes most differentially expressed during B-cell activation in this data set (Table 1). Coordination of these hub genes and separation from well-established regulators of germinal centre function (distributed in B1) suggest that *CCL22* and *CCL17* may represent a temporally separable early stage component of B-cell activation and preparation for CSR. Cluster A1 notably also contained *NFKB1* and *NFKB2*, encoding the NF-kB precursors p100 and p105, which together form higher order complexes to regulate the NF-kB signalling system^30^. Consistent with a hypothesis of shared regulatory control, cluster A1 was significantly enriched for transcription factor binding sites for Bach1 and Bach2 (*P*-values 6.29E-03 and 2.80E-02 respectively). Bach1 and Bach2 both promote B-cell development^31^ whilst Bach2 is also known to be critical for both CSR and somatic hyper-mutation^32^.

### Physical co-localisation of differentially expressed genes

Amongst the 1,399 differentially expressed TCs there were 78 physically co-localised groups, where co-localisation was defined as physical co-occurrence within 5 Kb. The size of these groups ranged between 2 and 6 TCs, with the majority (70 groups) containing 2 TCs, 6 groups containing 3, 1 group containing 4 and 1 group containing 6. The latter group (containing annotated members *HIST1H1A*, *HIST1H3A*, *HIST1H4A*, *HIST1H4B*, *HIST1H3B* and *HIST1H2AB*) physically localised to histone gene cluster 1 (HIST1) on chromosome 6p22-p21 with all members belonging to temporal co-expression cluster C4 indicating co-ordinated transcription. Histones are the major protein constituent of chromatin around which eukaryotic DNA is wound. H1, H2A, H3 and H4 are all replication-dependent histones, such that transcription is tightly linked to cell cycle phase, allowing packaging of newly replicated DNA. A second physically co-localised group of replication-dependent histones mapped to histone gene cluster 2 (HIST2) on chromosome 1q21 (3 member group: *HIST2H2BF*, *HIST2H2BE* and *HIST2H2AB*). A further 3 member co-localisation group on chromosome 11q12-q13.1 mapped to the evolutionarily conserved fatty acid desaturase (*FADS*) gene cluster, including poly-unsaturated fatty acid processing enzymes *FADS1* and *FADS2* and extending to the nearby flap structure-specific endonuclease 1 (*FEN1*). *FADS1* and *FADS2* have a head-to-head (H2H) orientation and control blood levels of polyunsaturated fatty acids (PUFA). Genes with a H2H orientation are prone to co-expression and co-functionality^33^. Disturbed expression of the *FADS* genes has previously been reported in several IgE-mediated diseases, including asthma^34^ and atopic dermatitis^35^,^36^, but this is the first such association with the direct cellular processes leading to IgE production. Variation in *FADS* gene expression during the activation time course is shown in supplementary Figure S3.

### Temporal changes in exon retention during B-cell activation

In order to catalogue variation in exon usage during B-cell activation via CD40 and IL-4 we examined the same longitudinal data set at the level of the exon (probeset) using Limma’s *diffSplice* function^37^. This method does not rely on existing gene annotations and therefore enables the detection of novel as well as established splice events. Differential exon splicing was non-uniformly distributed across the 288 hours following co-stimulation. An immediate early peak in exon splicing was seen in the 0–12 hour time point; 154 genes, of which only 79 (51.3%) attained significance for differential gene expression. A lesser peak was seen in the 120–288 hour time window; 50 genes, of which 29 (58.0%) attained significance for differential gene expression. Only a small number of genes achieved significance for differential splicing in the intervening periods (12–36 hours N = 3; 36–72 hours N = 12; and 72–120 hours N = 2). A list of the genes exhibiting the most significant variation in exon usage during the activation time course is given in Table 3.

In total just 110 (7.86%) of genes that met criteria for differential expression also achieved significance for differential splicing. Likewise, nearly half (49.31%) of differentially spliced genes showed no significant evidence of differential expression. These results demonstrate that the data gained through exon and transcript level expression profiling are incompletely overlapping and as such may offer complementary insights.

Patterns were sought amongst differentially spliced genes by testing for enrichment of gene ontology (GO) terms. At an adjusted *P*-value threshold of 0.05, significant enrichment was seen in the immediate early phase (0–12 hours, 5 terms), and the late phase (120–288 hour, 47 terms) only (Table S1). Genes demonstrating early changes to gene structure were most significantly enriched for molecular function terms relating to signalling and receptor function, including GO:0004871~*signal transducer activity (P*-value 4.27E-05, fold enrichment 2.30), GO:0060089~*molecular transducer activity (P*-value 4.27E-05, fold enrichment 2.30) and GO:0004872~*receptor activity (P*-value 6.00E-05, fold enrichment 2.58). Conversely, the most significantly enriched GO terms in genes showing late phase differences in exon retention related to cellular localisation in the *kinetochore* (GO:0000776, *P*-value 5.43E-05, fold enrichment 27.57) and assembly related biological processes including GO:0006334~*nucleosome assembly (P*-value 5.66E-05, fold enrichment 32.62) and GO:0031497~*chromatin assembly (P*-value 6.74E-05, fold enrichment 30.94).

**Figure 5:**
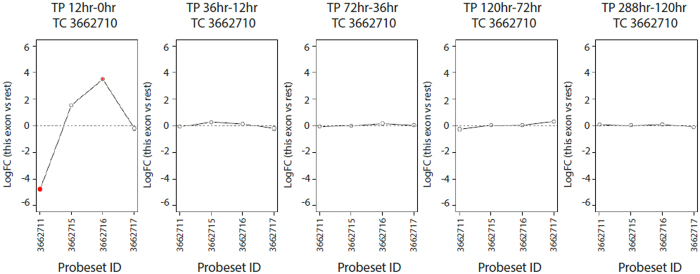
Differential *CCL17* exon retention during the activation of CSR. Relative log2-fold-changes between sequential time points are shown by exon for the gene *CCL17* (TC 3662710). Each exon is represented by a solid circle the size of which is weighted by its significance. Exons that meet criteria for significant differential splicing at a 5% FDR are highlighted in red. Relative log2-fold-change is defined as the difference between the log2-fold-change for a given exon and the overall log2-fold-change for that gene. Abbreviations: Transcript Cluster (TC), Fold Change (FC), Time Point (TP), False Discovery Rate (FDR).

The gene *XBP1* (TC 3956589), which has a well-established role in plasma cell differentiation, is known to undergo a splice reaction to generate its active form^38^,^39^. Here we observe a significant change in *XBP1* exon usage between 0 and 12-hours (FDR 5.30E-04), affecting probeset (exon) 3956608, which is relatively depleted (Figure S4). Among genes exhibiting the most significant variation in exon retention during the B-cell activation, we identified *CCL17* (FDR 2.33E-10), a hub in activation-related transcriptional cluster A1 outlined above. Two *CCL17* probesets showed differential usage between 0 and 12 hours: probeset 3662711, which was depleted relative to other exons, and probeset 3662716, which was relatively enriched (Fig. 5). Current Netaffx annotations place these probesets at exon 1 and exon 4 of *CCL17* respectively. Other top hits included the Semaphorin *SEMA4C* (FDR 5.97E-07, TC 2565592) with known effects on myogenic differentiation and emerging evidence of a role in B cell immune response^40^, and the protein phosphatase 1 inhibitor *PPP1R14A* (FDR 8.35E-07, TC 3861272) a gene capable of binding NF-kB and implicated in the control of airway hyper-responsiveness^41^.

### Stability of exon splicing reactions

Amongst differentially spliced genes, only 4 showed significant variation in 2 temporal windows. For 3 of these genes (hedgehog acyltransferase [*HHAT*, TC 2378369, probeset 2378431], chromatin licensing and DNA replication factor 1 [*CDT1*, TC 3673684, probeset 3673705] and the NDC80 kinetochore complex component *SPC24* [TC 3850660, probeset 3850667]) the same exon was affected in both windows, with opposite directions of effect relative to the remainder of the gene, suggesting a short-lived temporary change to exon structure in these genes. The remainder of differentially spliced genes achieved significance in only one time window, indicating that the majority of activation-related changes in exon retention are persistent, at least within the bounds of the time frame tested.

The outer kinetochore NDC80 complex consists of 4 proteins encoded by the genes *NDC80*, *NUF2*, *SPC24* and *SPC25*. As well as differential splicing of *SPC24* described above, all four genes met criteria for overall differential expression (Figure S5). The NDC80 complex is required for kinetochore assembly and chromosome congression, and participates in spindle checkpoint signalling^42^. These roles are vital to metaphase–anaphase transition during mitosis, and highlight a role in cell cycle control.

## Discussion

Immunoglobulin class switching occurs in mature B cells in response to antigen stimulation and co-stimulatory signals received from T helper cells. A limited number of B cell specific components have been identified as contributing to this process, notably AID encoded by the gene *AICDA*^43^, which initiates and is essential for not only CSR but also the related diversification event, somatic hypermutation^44^. Although class switching is essential for the expression of a different immunoglobulin isotype, it is not the only process required. Activated B cells undergo a coordinated program of proliferation, DNA recombination and mutation, rescue from apoptosis and cell differentiation in order to become antibody secreting plasma cells. Most insights into the mechanics of this process have been acquired through mice expressing reporter proteins or mice possessing a targeted genetic modification. Here we sought to explore the dynamic transcriptional processes underlying the germinal centre activities that accompany class switching to IgE and IgG through global exon profiling of a primary human B-cell time series following activation mimicking a Th2 mediated response. The data generated identify a host of novel CSR components that are known to individually regulate CSR, cell differentiation, proliferation and survival, and shed light on the temporal patterning and co-ordination between existing GC associated genes. Defining these genes’ roles in the activation of B cells leading to IgE production will not only help to understand the wider mechanisms of class switching, but may also provide insights into defective IgE responses and allergic pathogenesis.

We observed a complex transcriptional cascade composed of 1,399 genes during B-cell activation, organised at a fundamental level into 13 quantitative transcriptional profiles or trajectories. We confirmed involvement of several key germinal centre response genes including *AICDA*, *IRF4*, *XBP1*, *BATF3* and *NFIL3*, and showed that these well-established regulators of CSR exhibit synchronic, co-ordinated expression, forming a single 126 gene cluster, with *BHLHE40;* an environmentally inducible moderator of circadian rhythms and cellular differentiation, at its core. Consistent with *BHLHE40*’s status as a hub gene, *BHLHE40* was recently shown to operate as a master regulator of germinal centre activities, modulating the expression of >100 target genes^45^. Circadian oscillations in symptom severity are a prominent feature of atopic diseases including atopic dermatitis, asthma, chronic urticaria and allergic rhinitis^46^,^47^,^48^. Moreover, time-of-day related variation in IgE/mast cell allergic reactions was recently demonstrated to depend on the circadian clock in mice^49^, with such reactions pharmacologically inhibited through experimental adjustment of the molecular clock^50^. Mice deficient for the *BHLHE40* ortholog display a variety of immune features including abnormal IgG1 and IgE levels and defective elimination of activated B-cells, as well as exhibiting circadian rhythm phenomena^51^. Like *BHLHE40*, *NFIL3* also participates in signalling pathways relating to the circadian clock^52^ and together these data suggest there may be a circadian component to class switch recombination and that this may be of relevance to time-of-day phenomena in IgE driven diseases.

During the early stages of B cell activation the most pronounced changes in gene transcription affected the genes encoding the chemokines CCL22 and CCL17 and the cytokine receptor IL17RB. *CCL22* in particular showed a greater than 60-fold increase during the first 12 hours of activation. *CCL22* and *CCL17* are positioned at the very core of a single B-cell transcriptional profile, enriched for motifs targeting the Bach family of transcription factors, and temporally separable from the well-established GC related genes described above. *IL17RB* on the other hand, shows highly synchronic expression with *AICDA*, possessing one of the most proximal expression profiles of all genes examined. Clinically, both CCL22 and CCL17 have been suggested as biomarkers for disease activity in atopic dermatitis (AD), and raised cord blood (CB) levels of CCL22 predict subsequent allergic sensitisation, whilst raised CB CCL17 predicts the later development of allergic symptoms, including asthma^53^. Consistent with these observations, allergen exposure in sensitised individuals leads to a dynamic increase in CCL17 and CCL22^54^; an effect that can be inhibited *in vitro* through CCR4 blockade^55^. The secretion of CCL22 and CCL17 by B cells is well known to recruit Th2 cells (via CCR4) and as such is central to the germinal center response^56^. Similarly, CCR4 expression has previously been detected in non-germinal center B cells^57^. To our knowledge the importance of B cell expressed CCR4 in the human germinal center response has however not previously been investigated. These findings have important clinical implications given that high affinity neutraligands have been developed for CCL22 and CCL17, and these inhibit both the chemokine-induced intracellular calcium responses and CCR4 endocytosis *in vitro*, and inflammation *in vivo* in a murine model of asthma^58^. Of relevance to atopic dermatitis, the synthetic molecule 5,6-dihydroergosteol-glucoside (DHE-Glc) has also been shown to suppress TNF-α/IFN-γ-induced expression of *CCL17* and *CCL22* in the human keratinocyte cell line HaCaT, and attenuate levels of CCL22, CCL17 and IgE in a mouse model of atopic dermatitis as well as improve skin inflammatory symptoms^59^.

*IL17RB* abundance has previously been shown to increase upon allergen challenge in patients with seasonal allergic rhinitis^60^, and to correlate with IgE abundance in asthmatics, accounting for as much as 12% of its variance^61^. The data presented here further support the association and show that the increase in *IL17RB* forms an early component of the transcriptional cascade that initiates and the germinal centre response in B cells. Consistent with this observation, mice lacking the murine homolog of *IL17RB* show reduced levels of IgE^62^ and are resistant to IgE-mediated experimental food allergy^63^.

In addition to temporal clustering, B-cell activation-related genes showed evidence of physical clustering. The largest group included 6 TCs in evolutionarily conserved histone cluster 1, including a member of the H1 family, *HIST1H1A*. Histone H1 has previously been shown to influence mast cell-mediated type I hyperreactivity in mice^64^. Other physically co-localised groups included the fatty acid desaturase (*FADS*) gene cluster on chromosome 11q12. Here we showed that *FADS1* and *2* are induced early during the activation process. Altered expression of *FADS* genes has been documented in asthma and atopic dermatitis, and *FADS* gene cluster variants are suggested to modulate the effect of dietary intake on allergic disease^65^.

As well as gross changes in transcript abundance we have observed activation induced changes in exon usage, predominantly occurring early in the germinal centre response and sustained thereafter. Only three genes showed transient changes in exon usage, *HHAT*, *CDT1* and *SPC24*. The cell cycle regulatory protein CDT1 controls the initiation of DNA replication through its interactions with Geminin, and shows rapid recruitment to sites of DNA damage in human cells^66^; suggesting a potential contribution to CSR at the point of DNA double-strand break initiation and repair. *SPC24* forms part of the NDC80 kinetochore complex. This complex is encoded by 4 genes, all of which were differentially expressed during activation. Several NDC80 components have been implicated in cancer prognosis, with overexpression exerting a positive impact on cellular proliferation, an effect that is at least partially reversible through experimental knockdown^67^,^68^. A host of other genes with known alternative transcripts showed differential exon usage during the culture time course, and future functional distinction between these isoforms may yield novel insights into B-cell germinal centre biology.

Finally, the data presented reveal involvement of various innate immunity candidate genes, associated with diseases such as asthma and atopic dermatitis, in the fundamental mechanisms of B-cell activation and class switch recombination. A group of these genes including *NOD2*, *SOCS1* and *LTA* show synchronic expression with key components of CSR and germinal centre activity such as *AICDA* and *NFIL3*. Further exploration of these patterns may provide novel insights into the molecular mechanism underlying their involvement in disease.

In summary, we present a complex transcriptional cascade occurring following activation of germinal centre activities in primary human tonsillar B-cells. We acknowledge that these transcriptional phenomena may be subject to variation relating to the location of B-cell activation and the prevailing microenvironment. Nevertheless, these data provide a solid foundation upon which future investigations may build. We show that *CCL22*, *CCL17* and *IL17RB* play central roles in the early stages of B-cell activation, and that *CCL22* and *CCL17* exhibit a quantitative profile distinct from several established mediators of the germinal centre response. We additionally demonstrate that B-cell activation coincides with systematic variation in exon usage, affecting a spectrum of targets, including notably *CCL17*. Understanding these genes’ contribution to germinal centre activities will not only bring new insights of the mechanisms of class switching but may also provide novel opportunities for therapeutic intervention.

## Methods

The study was approved by the ethics committee of Guy’s Hospital, London and was performed in accordance with the approved guidelines and regulations.

### Naive B cells isolation

Human B cells were isolated from the tonsils of three patients undergoing routine tonsillectomies at the Evelina Children’s Hospital. All donors were recruited in accordance with the guidelines imposed by Guy’s Hospital Research Ethics Committee (study No. 08 H0804 94) and informed consent was obtained before inclusion in the study. All research protocols were approved (following review) by Guy’s and St. Thomas’ Foundation Trust Research and Development Dept. (Study No. RJ1 09/0325). The patients were all aged between 2 and 14 years, had no history of asthma or any known allergies or long standing medical conditions (except tonsillitis) and were not taking any medications. The patients’ parents or legal guardians gave informed written consent for participation in this study. Total B cells were isolated from the tonsil as previously described^69^. Naïve B cells were subsequently separated by FACSorting (FACSAria™, BD Biosciences), Naïve B cells being IgD^+^, CD38^Low^, CD27^−^. B cell purity was assessed by flow cytometry using fluorescently-labelled antibodies (Dako Cytomation) and a FACSCalibur™ flow cytometer (BD Biosciences)^4^.

### Cell culture

B cells were cultured in 24-well plates (Nunc) at 0.5 × 10^6 ^cells/mL in RPMI medium (Invitrogen Ltd.), supplemented with transferrin (35 μg/mL, Sigma-Aldrich Company Ltd.), insulin (5 μg/mL, Sigma-Aldrich Company Ltd.), penicillin (100 IU/mL), streptomycin (100 μg/mL), glutamine (2 mM) (all Invitrogen Ltd.) and 10% foetal bovine serum (FBS) (Hyclone, Perbio Biosciences Ltd.). To stimulate CSR to IgE, media was supplemented with 1 μg/mL anti-CD40 antibody (G28.5, ATCC) and 200 IU/mL of recombinant human IL-4 (R&D Systems Ltd.)^4^.

### RNA extraction and expression profiling

Post stimulation with IL-4 and anti-CD40, cells were harvested at 0, 12, 36, 72, 120 and 288 hours. RNA was extracted using the RNeasy MINI Kit (Qiagen Ltd.). Quantity and quality of RNA was assessed using a spectrophotometer and Bioanalyser RNA Nano Chip (Agilent Technologies Ltd) respectively. One μg of high quality RNA was synthesised to double-stranded cDNA using the one-cycle cDNA kit (Affymetrix Ltd.) after RNA reduction to reduce 18 S RNA. *In vitro* transcription of cRNA was carried out using the IVT kit (Affymetrix Ltd.) resulting in complementary amplification and biotin labelling. cRNA was purified, concentrated and checked for quality and quantity. Ten μg of cRNA was fragmented and hybridised onto a Human Exon 1.0 ST Array (Affymetrix Ltd) for 16 hours. GeneChips were washed, stained on a Fluidics 450 station and scanned using a high-resolution scanner (Affymetrix Ltd.).

### Array data analysis

All microarray data analysis was carried out in accordance with the recommended best practices in the field. Data quality was assessed using Affymetrix Power Tools (APT, 1.16.1) and Array Quality Metrics (Version 3.22.1)^70^. Subsequent analyses were carried out both at the exon (probeset) and the gene (meta-probeset) level for the characterisation of dynamics in exon retention and global gene expression respectively.

#### I. Exon level analysis

Raw signal intensity data were normalised and summarized into core probesets through the Robust Multi-array Average (RMA) method implemented in APT. Probesets failing to achieve a significant DABG statistic in any sample, and probesets annotated as cross hybridising (Release 35 Affymetrix annotations) were removed. Following filtration 183,349 probesets, relating to 16,282 genes with >1 exon were retained for analysis.

Evidence for variation in exon usage was sought using the Limma function *diffSplice* (Version 3.22.7) comparing log-fold changes between sequential time points for exons of the same gene, and yielding gene-wise Simes adjusted *P*-values, with the false discovery rate (FDR) controlled under 0.05^25^.

Patterns of functional or cellular enrichment were assessed though Gene Ontology (GO) using Database for Annotation, Visualization and Integrated Discovery (DAVID)(v6.7)^71^,^72^. Terms relating to molecular function (MF), biological process (BP) and cellular component (CC) were tested for enrichment in the differentially spliced genes relative to all genes targeted by filtered probesets. Significance was assessed using a Benjamini adjusted *P*-value to control the family-wide false discovery rate (FDR) under 0.05.

#### II. Gene level analysis

Raw signal intensity data were normalised and summarized into core transcript clusters (TCs) using the APT implementation of the RMA algorithm. TCs classified as part of the main design were retained. Since DABG is not suitable for analyses performed at the gene level, presence/absence was inferred directly from expression values, with universally low expressed TCs removed. Universal low expression was defined as expression less than or equal to the dataset median across all samples in the study.

Differential expression analysis was performed in Limma, blocking by sample, generating comparisons between each sequential time point and calculating an overall test of significance for each gene (the moderated F statistic). *P*-values were adjusted for multiple testing by the Benjamini and Hochberg method, controlling the expected FDR below 0.05. Patterns of enrichment for Gene Ontology terms were assessed though DAVID (v6.7) using the Benjamini method of *P*-value adjustment, relative to all genes (TC) tested for differential expression. Temporal clustering patterns were determined by fuzzy c-means clustering implemented in Mfuzz (2.26.0)^73^. Sub-structure within temporal profiles was assessed through hierarchical clustering using the complete linkage method and the Euclidean distance metric. A cut height of 4 was applied to allow differential colouring of dendrogram branches. Genomic co-localisation of differentially expressed genes was examined by seeking differentially expressed genes located within 5 Kb of one another. Cluster-specific enrichment for UCSC transcription factor binding sites and GO Biological Process terms was tested using DAVID and the Benjamini method of *P*-value adjustment relative to all genes (transcript clusters) tested for differential expression^72^,^73^.

Microarray data for samples used in the comparison have been deposited in ArrayExpress with Accession Number E-MTAB-4937.

## Additional Information

**How to cite this article**: Zhang, Y. *et al.* Global gene regulation during activation of immunoglobulin class switching in human B cells. *Sci. Rep.* **6**, 37988; doi: 10.1038/srep37988 (2016).

**Publisher's note:** Springer Nature remains neutral with regard to jurisdictional claims in published maps and institutional affiliations.

## Supplementary Material

Supplementary Information

## Figures and Tables

**Table 1:** The top 20 differentially expressed TC across the 288-hour time course.

Transcript cluster	F	P-Value	Adj.P.Val	Chromosome	*Gene assignment*
3662687	97.57	3.94E-11	4.39E-07	chr16	***CCL22***
2624565	80.50	1.64E-10	9.16E-07	chr3	***IL17RB***
3662710	76.11	2.49E-10	9.24E-07	chr16	***CCL17***
3214451	65.55	7.45E-10	2.08E-06	chr9	***NFIL3***
2450345	63.20	9.74E-10	2.17E-06	chr1	***KIF14***
3235789	56.97	2.07E-09	3.33E-06	chr10	***MCM10***
3639031	56.90	2.09E-09	3.33E-06	chr15	***PRC1***
3223738	55.01	2.68E-09	3.47E-06	chr9	***TRAF1***
2899102	54.66	2.80E-09	3.47E-06	chr6	**—**
3776139	51.59	4.25E-09	4.56E-06	chr18	***NDC80***
2364438	50.73	4.81E-09	4.56E-06	chr1	***NUF2***
3636391	50.09	5.26E-09	4.56E-06	chr15	****HOMER2/*LOC100131860*/HOMER2P1****
2616131	50.02	5.32E-09	4.56E-06	chr3	****CCR4/SEC13P1****
2838656	49.26	5.94E-09	4.72E-06	chr5	***HMMR***
2417528	48.72	6.42E-09	4.77E-06	chr1	***DEPDC1***
3910785	48.01	7.14E-09	4.89E-06	chr20	****AURKA/*AURKAPS1*/RAB3GAP2****
3587457	47.72	7.46E-09	4.89E-06	chr15	****ARHGAP11B/*ARHGAP11A*/LOC100288637****
2946232	45.73	1.01E-08	6.27E-06	chr6	***HIST1H1C***
2792166	44.96	1.14E-08	6.49E-06	chr4	***MARCH1***

**Table 2:** Temporal cluster summary.

Cluster	# Members	Core genes (Gene ID, Membership)	GO Biological Process enrichment (Benjamini P-value)	TFBS enrichment (Benjamini P-value)
A1	153	*CCL22* (3662687, 0.97), *CCL17* (3662710, 0.96)	NS	BACH1 (6.29E-03), BACH2 (2.80E-02)
A2	83	*TMEM2* (3209384, 0.99), *TRIB2* (2470165, 0.99)	GO:0002376~immune system process (2.35E-03)	NS
B1	126	*BHLHE40* (2608725, 0.95), *AARS* (3697015, 0.92)	GO:0044106~cellular amine metabolic process (2.01E-02)	RSRFC4 (1.86E-02), STAT (1.71E-02)
B2	112	*ZNF581* (3842301, 0.95), *GAS6* (3502829, 0.92)	NS	NS
C1	79	*MRTO4* (2323559, 0.98), *CLUH* (3741171, 0.97)	GO:0022613~ribonucleoprotein complex biogenesis (2.78E-04)	NS
C2	112	*GNL3* (2624074, 0.96), *ATP5B* (3458033, 0.96)	NS	NS
C3	105	*DTL* (2378937, 0.96), *CLSPN* (2406420, 0.93)	GO:0006259~DNA metabolic process (2.79E-47)	NS
C4	151	*BUB1B* (3589697, 0.98), *KIF2C* (2334098, 0.98)	GO:0000279~M phase (2.94E-52)	NFY (1.39E-05)
C5	99	*PGRMC1* (3988740, 0.94), *VAT1L* (3669552, 0.86)	GO:0022403~cell cycle phase (1.12E-03)	NS
C6	128	*HMGCL* (2401609, 0.93), *FLOT1* (2948587, 0.89)	NS	NS
D1	69	*OAS1* (3432438, 0.98), *CBLB* (2687255, 0.96)	NS	NS
D2	69	*JMJD7* (3590709, 0.93), *CALCOCO1* (3456353, 0.91)	NS	NS
D3	113	*LRIF1* (2427688, 0.94), *APOBEC3H* (3945684, 0.92)	NS	NS
Sum	1399			

TFBS significant at a 5% threshold are shown. Where more than one GO term achieves significance at this threshold, the term accompanied by the lowest Benjamini P-value is shown. The two genes exhibiting the highest membership values for a given cluster are reported.

Abbreviations: Not Significant (NS), Transcription Factor Binding Site (TFBS), Gene Ontology (GO), Biological Process (BP).

**Table 3:** Uniquely annotated genes showing the most significant evidence of differential splicing at a 5% FDR.

TC	P Value	FDR	Chr	Gene Assignment	Time Window
2316605	9.13E-19	7.43E-15	1	*PLCH2*	TP.12 hr-TP.0 hr
3735847	4.77E-16	2.59E-12	17	*SEPT9*	TP.12 hr-TP.0 hr
3662710	5.73E-14	2.33E-10	16	*CCL17*	TP.12 hr-TP.0 hr
3368940	9.91E-13	3.23E-09	11	*ABTB2*	TP.12 hr-TP.0 hr
3740432	2.64E-10	5.97E-07	17	*SCARF1*	TP.12 hr-TP.0 hr
2565592	2.79E-10	5.97E-07	2	*SEMA4C*	TP.12 hr-TP.0 hr
2739714	2.93E-10	5.97E-07	4	*C4orf32*	TP.12 hr-TP.0 hr
3861272	4.62E-10	8.35E-07	19	*PPP1R14A*	TP.12 hr-TP.0 hr
3818515	7.89E-10	1.22E-06	19	*TRIP10*	TP.12 hr-TP.0 hr
3866094	8.26E-10	1.22E-06	19	*PTGIR*	TP.12 hr-TP.0 hr
3850660	1.10E-08	1.79E-04	19	*SPC24*	TP.36 hr-TP.12 hr
3957873	2.41E-07	1.96E-03	22	*EIF4ENIF1*	TP.36 hr-TP.12 hr
3204692	2.91E-06	1.58E-02	9	*ARHGEF39*	TP.36 hr-TP.12 hr
3585905	2.22E-06	1.21E-02	15	*APBA2*	TP.72 hr-TP.36 hr
3852407	5.92E-06	1.91E-02	19	*RFX1*	TP.72 hr-TP.36 hr
2327391	8.19E-06	1.91E-02	1	*SESN2*	TP.72 hr-TP.36 hr
3590014	8.23E-06	1.91E-02	15	*CASC5*	TP.72 hr-TP.36 hr
2887490	1.33E-05	2.71E-02	5	*STC2*	TP.72 hr-TP.36 hr
3838254	1.62E-05	2.89E-02	19	*PPFIA3*	TP.72 hr-TP.36 hr
2895792	1.78E-05	2.89E-02	6	*RNF182*	TP.72 hr-TP.36 hr
2827772	2.69E-05	3.98E-02	5	*ADAMTS19*	TP.72 hr-TP.36 hr
3184896	1.75E-06	1.73E-02	9	*ZNF483*	TP.120 hr-TP.72 hr
3514348	2.13E-06	1.73E-02	13	*GUCY1B2*	TP.120 hr-TP.72 hr
3538403	6.88E-09	5.33E-05	14	*LRRC9*	TP.288 hr-TP.120 hr
3850660	9.82E-09	5.33E-05	19	*SPC24*	TP.288 hr-TP.120 hr
3673684	3.96E-08	1.61E-04	16	*CDT1*	TP.288 hr-TP.120 hr
2946369	6.89E-08	1.94E-04	6	—	TP.288 hr-TP.120 hr
2635184	7.14E-08	1.94E-04	3	*HHLA2*	TP.288 hr-TP.120 hr
3565663	2.92E-07	5.06E-04	14	*DLGAP5*	TP.288 hr-TP.120 hr
2334098	3.11E-07	5.06E-04	1	*KIF2C*	TP.288 hr-TP.120 hr
3296512	1.01E-06	1.50E-03	10	*POLR3A*	TP.288 hr-TP.120 hr
3903146	1.68E-06	1.96E-03	20	*E2F1*	TP.288 hr-TP.120 hr
3248289	2.57E-06	2.78E-03	10	*CDK1*	TP.288 hr-TP.120 hr

For ease of interpretation TC accompanied by more than one gene annotation are not shown. Results are limited to the top ten TC exceeding an FDR of 0.05, per time window. Abbreviations: Chromosome (Chr), Time Point (TP), Hours (hr), False Discovery Rate (FDR), Transcript Cluster (TC).
