# Supplementary material for: Global gene regulation during activation of immunoglobulin class switching in human B cells
Source: Sci Rep. 2016 Nov 29;6:37988. doi: 10.1038/srep37988 (PMC5126563; doi:10.1038/srep37988)
Supplement: Supplementary Information [file srep37988-s1.doc]

**Supplementary data:**

**Global gene regulation during activation of immunoglobulin class switching in human B cells**

Youming Zhang^1*^, David J Fear^2^, Saffron A G Willis-Owen^1^, William O Cookson^1^ and

Miriam F Moffatt^1^

^1^Molecular Genomics and Genetics Group, National Heart and Lung Institute, Imperial College, London SW3 6LY, UK

^2^Medical Research Council and Asthma UK Centre in Allergic Mechanisms of Asthma, King's College London, London, United Kingdom

#YZ, DJF, SAW contributed equally to the paper

*To whom correspondence should be addressed. Email: y.zhang@imperial.ac.uk

Figure S1: Volcano plots detailing differential gene expression within each sequential time window.

Expression is on a log2 scale.

Figure S2: Average minimum centroid distance across a range of temporal expression cluster numbers.

Figure S3: Expression of the two *FADS* genes, *FADS1* and *FADS2* during the activation of CSR.

Abundance is displayed on a log2 scale. Results of triplicates are shown. Time point is in hours. Abbreviations: Transcript Cluster (TC).

Figure S4: Differential retention of *XBP1* exons during the activation of CSR.

Relative log2-fold-changes between sequential time points are shown by exon for the gene *XBP1* (TC 3956589). Each exon is represented by a solid circle the size of which is weighted by its significance. Exons that meet criteria for significant differential splicing at a 5% FDR are highlighted in red. Relative log2-fold-change is defined as the difference between the log2-fold-change for a given exon and the overall log2-fold-change for that gene. Abbreviations: Transcript Cluster (TC), Fold Change (FC), Time Point (TP), False Discovery Rate (FDR)

Figure S5: Expression of four genes encoding the outer kinetochore NDC80 complex during the activation of CSR.

Abundance is displayed on a log2 scale. Results of triplicates are shown. Time point is in hours. Abbreviations: Transcript Cluster (TC).

Table S1: Gene Ontology terms significantly enriched in genes showing evidence of differential splicing

| ***GO***  ***category*** | ***GO term*** | ***Fold Enrichment*** | ***Benjamini P-value*** | ***Time window*** |
| --- | --- | --- | --- | --- |
| MF | GO:0004871~signal transducer activity | **2.30** | **4.27E-05** | 0-12hr |
| MF | GO:0060089~molecular transducer activity | **2.30** | **4.27E-05** | 0-12hr |
| MF | GO:0004872~receptor activity | **2.58** | **6.00E-05** | 0-12hr |
| MF | GO:0004888~transmembrane receptor activity | **2.70** | **2.60E-03** | 0-12hr |
| MF | GO:0005044~scavenger receptor activity | **12.87** | **8.98E-03** | 0-12hr |
| BP | GO:0022403~cell cycle phase | **10.85** | **7.84E-08** | 120-288hr |
| BP | GO:0000087~M phase of mitotic cell cycle | **15.97** | **1.20E-07** | 120-288hr |
| BP | GO:0048285~organelle fission | **15.59** | **1.21E-07** | 120-288hr |
| BP | GO:0000280~nuclear division | **16.20** | **1.38E-07** | 120-288hr |
| BP | GO:0007067~mitosis | **16.20** | **1.38E-07** | 120-288hr |
| BP | GO:0000278~mitotic cell cycle | **11.33** | **1.54E-07** | 120-288hr |
| BP | GO:0007049~cell cycle | **6.71** | **2.08E-07** | 120-288hr |
| BP | GO:0000279~M phase | **11.84** | **2.14E-07** | 120-288hr |
| BP | GO:0022402~cell cycle process | **7.96** | **4.34E-07** | 120-288hr |
| BP | GO:0006996~organelle organization | **4.51** | **3.45E-06** | 120-288hr |
| CC | GO:0044427~chromosomal part | **10.80** | **4.24E-06** | 120-288hr |
| CC | GO:0005694~chromosome | **9.12** | **1.03E-05** | 120-288hr |
| CC | GO:0000777~condensed chromosome kinetochore | **36.76** | **2.13E-05** | 120-288hr |
| CC | GO:0000779~condensed chromosome, centromeric region | **32.02** | **3.21E-05** | 120-288hr |
| CC | GO:0000776~kinetochore | **27.57** | **5.43E-05** | 120-288hr |
| BP | GO:0006334~nucleosome assembly | **32.62** | **5.66E-05** | 120-288hr |
| BP | GO:0031497~chromatin assembly | **30.94** | **6.74E-05** | 120-288hr |
| CC | GO:0005819~spindle | **16.54** | **6.74E-05** | 120-288hr |
| BP | GO:0065004~protein-DNA complex assembly | **29.41** | **7.96E-05** | 120-288hr |
| BP | GO:0034728~nucleosome organization | **28.94** | **7.97E-05** | 120-288hr |
| CC | GO:0000786~nucleosome | **43.54** | **8.34E-05** | 120-288hr |
| BP | GO:0051301~cell division | **9.82** | **1.04E-04** | 120-288hr |
| CC | GO:0044446~intracellular organelle part | **2.26** | **1.38E-04** | 120-288hr |
| CC | GO:0044422~organelle part | **2.24** | **1.40E-04** | 120-288hr |
| CC | GO:0043232~intracellular non-membrane-bounded organelle | **2.90** | **1.58E-04** | 120-288hr |
| CC | GO:0043228~non-membrane-bounded organelle | **2.90** | **1.58E-04** | 120-288hr |
| CC | GO:0005634~nucleus | **2.05** | **1.63E-04** | 120-288hr |
| BP | GO:0016043~cellular component organization | **2.80** | **1.75E-04** | 120-288hr |
| CC | GO:0000775~chromosome, centromeric region | **17.11** | **2.36E-04** | 120-288hr |
| CC | GO:0000793~condensed chromosome | **16.14** | **2.69E-04** | 120-288hr |
| CC | GO:0032993~protein-DNA complex | **27.57** | **2.83E-04** | 120-288hr |
| BP | GO:0006323~DNA packaging | **20.86** | **3.29E-04** | 120-288hr |
| CC | GO:0031262~Ndc80 complex | **248.15** | **4.47E-04** | 120-288hr |
| BP | GO:0006333~chromatin assembly or disassembly | **19.29** | **4.55E-04** | 120-288hr |
| BP | GO:0051276~chromosome organization | **6.33** | **1.98E-03** | 120-288hr |
| BP | GO:0007346~regulation of mitotic cell cycle | **12.29** | **3.56E-03** | 120-288hr |
| CC | GO:0015630~microtubule cytoskeleton | **5.38** | **4.21E-03** | 120-288hr |
| CC | GO:0032991~macromolecular complex | **2.26** | **4.71E-03** | 120-288hr |
| CC | GO:0000785~chromatin | **10.09** | **9.75E-03** | 120-288hr |
| BP | GO:0034621~cellular macromolecular complex subunit organization | **7.22** | **1.03E-02** | 120-288hr |
| BP | GO:0051726~regulation of cell cycle | **6.71** | **1.45E-02** | 120-288hr |
| BP | GO:0006325~chromatin organization | **6.42** | **1.75E-02** | 120-288hr |
| CC | GO:0043227~membrane-bounded organelle | **1.43** | **2.33E-02** | 120-288hr |
| CC | GO:0043231~intracellular membrane-bounded organelle | **1.43** | **2.40E-02** | 120-288hr |
| BP | GO:0034622~cellular macromolecular complex assembly | **7.04** | **3.74E-02** | 120-288hr |
| CC | GO:0044430~cytoskeletal part | **3.43** | **4.09E-02** | 120-288hr |
| CC | GO:0005654~nucleoplasm | **3.28** | **4.99E-02** | 120-288hr |

Abbreviations: Hours (hr), Cellular Component (CC), Biological Process (BP), Molecular Function (MF), Gene Ontology (GO), False Discovery Rate (FDR).
